# Therapeutic options in rotator cuff calcific tendinopathy

**DOI:** 10.1051/sicotj/2025003

**Published:** 2025-02-20

**Authors:** Daniel Moya, Mustafa Rashid, Sergio Rowinski, Saad Al-Qahtani, Pedro Bernáldez Domínguez, Diego Gómez, Ignacio Dallo

**Affiliations:** 1 Department of Orthopedic Surgery, Hospital Británico de Buenos Aires C1280 AEB Argentina; 2 Manchester University NHS Foundation Trust Manchester M13 9WL United Kindom; 3 Shoulder Planet Clínica Ortopédica São Paulo 01258-010 Brazil; 4 Department of Orthopedic Surgery, College of Medicine, Imam Abdulrahman Bin Faisal University Dammam 31441 Saudi Arabia; 5 SportsMe Medical Center Seville 41013 Spain

**Keywords:** Rotator cuff, Calcific tendinopathy, Shock waves, ESWT, Ultrasound-guided barbotage

## Abstract

There are many variables that influence the decision-making process in the treatment of rotator cuff calcifications. The stage of the deposit, prognostic factors, previous failed treatments, pain level, and functional disability must all be considered. The tendency for spontaneous resolution is an important reason to always exhaust conservative treatment, being non-invasive options the first line of treatment. The emergence of focused shock wave therapy offered a powerful tool for the non-invasive management of rotator cuff calcifications. High-energy focused shock waves have a high degree of recommendation for the treatment of rotator cuff calcifications, supported by meta-analyses and systematic reviews. If non-invasive techniques fail, there is the possibility of moving to a minimally invasive procedure such as ultrasound-guided barbotage. Finally, classic invasive techniques are also a frequent indication, including open surgery and arthroscopy. As each treatment has advantages and disadvantages, the most advisable strategy is to progress from the least invasive therapeutic methods to the most invasive ones without losing sight of the clinical stage of the disease and the general context of each patient.

## Introduction

Rotator cuff calcific tendinopathy (RCCT) is a condition characterized by the deposition of calcium hydroxyapatite crystals within the tendons of the rotator cuff. This tendinopathy is frequently found in the middle age of life with a slight predominance in women [1]. The most frequent location is in the supraspinatus tendon, but it can occur in any of the rotator cuff tendons [1].

This is an entity exposed to many controversies. Although several theories have been proposed, its exact pathogenesis is not yet clearly known [1–7]. The clinical features of this condition vary from being asymptomatic to acute episodes of intense pain [1, 3]. Its clinical course is often unpredictable. There is no consensus-accepted treatment algorithm [1].

The objective of this review is to analyze the different theories of RCCT etiology, describe the most frequently used therapeutic options, and discuss treatment decision-making.

## Aetiology

RCCT is an entity characterised by the presence of calcium deposits which appear as amorphous areas located within the tendon fibres. The presence of chondrocyte-like cells surrounding deposits with a rounded morphology, located in the lacuna are also characteristic [8].

The true aetiology of RCCT has not been conclusively determined. Hans-Klaus Uhthoff popularized a theory of calcific tendinitis aetiology, drawn from observations of biopsies of human rotator cuff tissue with calcific tendinopathy [2, 3]. He observed that the tendon demonstrated fibrocartilage with a predilection for calcification, similar to incomplete endochondral calcification, forming aggregates of crystals in extracellular vesicles [2]. Uhthoff also identified “phases” of calcific tendinitis (pre-calcific, formative, resorptive, and post-calcific) and correlated severe pain with the resorptive phase (characterised by presence of neovascularisation and phagocytosis) [3]. Crystals deposited in matrix vesicles coalesce in the formative phase, followed by a resting phase of inactivity, and may progress to a resorptive phase characterised by appearance of thin-walled vascular channels bringing macrophages, polymorphonuclear (PMN) cells, and fibroblasts that resorb the deposit over time [8].

There are indeed many theories as to the nature of RCCT, including degenerative calcification secondary to vascular ischaemia, repetitive microtrauma, and necrosis of tenocytes releasing intracellular calcium into the extracellular matrix [9]. However, a popular theory relates to a yet-to-be-identified aberrant inflammatory cascade that results in formation and deposition of calcium rather than resolution of inflammation and normal tendon homeostasis [6].

## Prevalence

Several studies have been reported on the presence of calcific deposits on shoulder imaging [1–3]. Many cases present asymptomatically. Indeed, when in clinically asymptomatic shoulders, rotator cuff calcium deposits are found in imaging, such calcific deposits are simply interpreted as “by-standers”, with no pathological value [7, 10, 11].

In a study including 304 asymptomatic female volunteers, 24% had calcific deposits on shoulder ultrasonography [7].

A similar study recruited 302 asymptomatic women attending a gynaecology clinic to participate in a screening questionnaire and ultrasound scan of both shoulders [10]. Of the total 604 shoulders, 103 had calcific deposits on ultrasonography (17.8%) [10]. One third of those with calcium deposits reported pain. Intrinsic factors correlated with pain where: supraspinatus location and whether multiple tendons were involved. Extrinsic factors correlated with pain included age and BMI (>25) [10]. Another study included 465 asymptomatic shoulders in women of working age [11]. Authors reported that 19% of dominant shoulders and 12% in non-dominant shoulders had calcific deposits on ultrasonography [11].

Several studies have also screened small samples of athletes participating in various sports [12–15]. They report the prevalence of calcific deposits in 17–31%. These studies are summarised in Table 1.

**Table 1:** Summary of studies reporting the prevalence of calcific tendinitis in various sporting populations.

Author	Population	Age + Gender	Overall prevalence	Symptomatic?
Brasseur et al. [12]	150 veteran tennis players participating in the French veteran championship)	85 men – mean age 57 (range 35–76); 65 women – mean age 52 (range 35–77)	42/150 dominant shoulder supraspinatus	22/72 supraspinatus calcific deposits have had or currently have pain
			30/150 non-dominant shoulder supraspinatus	14/32 subscapularis calcific deposits have had or currently have pain
			2/150 dominant shoulder infraspinatus	
			4/150 non-dominant shoulder infraspinatus	
			24/150 dominant shoulder subscapularis	
			8/150 non-dominant subscapularis	
Suzuki et al. [13]	40 competitive-level masters swimmers	15 males; 25 women	11/40 dominant shoulder supraspinatus	10/17 symptomatic with supraspinatus calcification
		Mean age 52 years (range 30–65 years)	6/40 non-dominant shoulder supraspinatus	15/31 symptomatic with supraspinatus calcification
			16/40 dominant shoulder subscapularis	
			15/40 non-dominant subscapularis	
Monteleone et al. [14]	26 competitive-level rugby players	Mean age 23; all male	8/26 supraspinatus	N/R
Navas-Mosqueda et al. [15]	30 professional bullfighters	Mean age 25; all male	1/30 dominant supraspinatus	N/R
			1/30 dominant infraspinatus; 2/30 non-dominant infraspinatus	
			5/30 dominant subscapularis	

## Natural history and spontaneous resolution

The natural history of RCCT is equally difficult to appreciate as most patients with calcific deposits are neither symptomatic nor likely to present to a healthcare provider [7, 10, 11]. The small subset of patients with symptomatic calcific tendinitis within the rotator cuff that fail to spontaneously resolve within a given time period, ultimately present to a physician or a physiotherapist. Harvie et al. [16] reported the results of 125 shoulders in 102 patients presenting to a shoulder surgeon’s outpatient clinic for treatment. In this cohort, 62% were treated non-operatively (73% satisfied with treatment); 38.4% failed non-operative treatment (85% satisfied with surgical intervention). They also reported that a high proportion of women were treated for a variety of endocrine or menstrual disorders (50/73). In this “endocrine group” (81 shoulders in 66 patients), 94% were women. Compared to the control group, their symptoms onset was earlier in life (41 years vs. 47 years), symptom duration was longer (80 months vs. 47 months), and they were more likely to fail non-operative management (47% vs 23%) [16].

## Conservative treatment

Conservative treatment usually includes rest, systemic anti-inflammatory drugs, subacromial steroid injections, physical therapy, manual therapy, electrotherapy, iontophoresis, and exercises [1, 17].

The tendency towards spontaneous resolution [1, 2, 18] supports the idea that conservative treatment must be exhausted before any surgical approach may be considered. It makes sense, even in episodes of great pain, as this can be the clinical manifestation of an acute resorption of the calcific deposit [1, 2].

Regarding chronic conditions, from 60% to 80% success rate have been reported with conservative treatment [17]. However, DePalma and Kruper [19] had 84% good results in the short term in patients treated conservatively; however being reevaluated after 1 year, the success rate fell to 61%. Many patients end up living with a chronic condition of pain with ups and downs, and limited mobility [18, 19].

Good patient selection can reduce poor outcomes. Ogon et al. [20] described factors that are indicators of worse results with conservative treatment, including simultaneous bilateral deposits, large deposits, projection of the calcification medially beyond the limit of the acromioclavicular joint and location in the anterior area of the acromion. Positive prognostic factors were a Gärtner type III deposit and a lack of sonographic sound extinction of the calcific deposit [20, 21].

There is no stipulated time period for considering conservative treatment formally exhausted [18]. It will depend on the patient’s tolerance to the painful condition and the degree of disability.

## Extracorporeal shockwaves treatment

**Figure 1:**
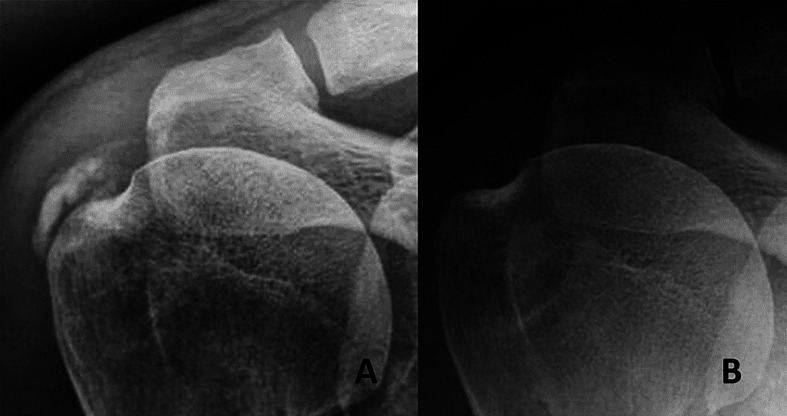
(A) AP view of a right subacromial space showing a Gärtner type II calcification located in the supraspinatus tendon. (B) The same X-ray projection after three sessions of focused shock waves.

The advent of shock wave therapy offered another powerful tool for the treatment of RCCT (Figure 1). Focused shock wave therapy is a non-invasive method with a good effectiveness rate and low chances of complications [18, 22, 23].

Unfortunately, there is a lot of confusion regarding this method [24]. In daily practice, and in part of the literature, focused waves and radial pressure waves are included as “Extracorporeal Shock Wawe Therapy” (ESWT). However, these two technologies differ in their generation devices, physical characteristics, and mechanism of action, but they share several indications [22, 24].

High-energy focused waves have a high degree of recommendation for the treatment of rotator cuff calcifications, supported by meta-analyses and systematic reviews [22]. Resorption rates of calcifications greater than 80% have been reported [23]. There are different theories about the mechanism of action of shock waves on rotator cuff calcifications [25], going from the physical effect as a result of cavitation, to complex physical and chemical reactions that generate a biological reaction [22]. Success rate may be lower in Gärtner type I calcifications [25]. In any case, even in cases without response, the use of shock wave treatment does not affect the final result of an eventual surgery [26].

Studies comparing the results of shock waves with open and arthroscopic surgery have reported comparable results at lower cost in the case of shock waves [27, 28].

On the other hand, in the case of radial pressure waves, there are isolated and contradictory reports, making its degree of recommendation low [22].

## Ultrasound-guided “barbotage”

**Figure 2:**
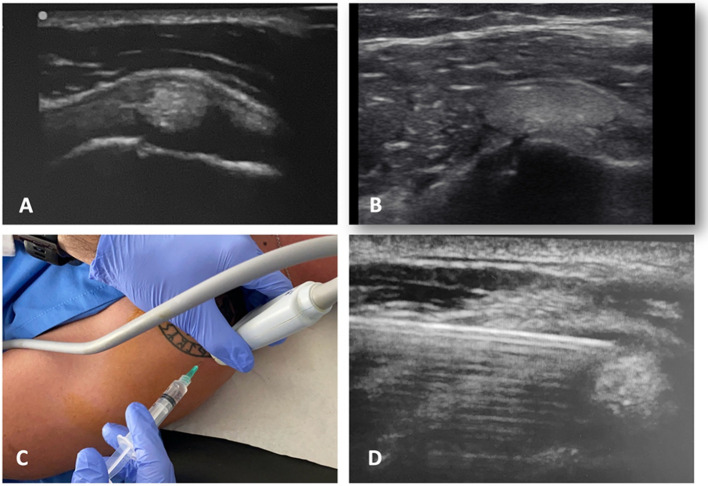
(A) Ultrasound image of a calcification in the supraspinatus tendon (short axis). (B) Ultrasound image of the long axis of the same case. (C) Ultrasound-guided barbotage technique. (D) Ultrasound image of the procedure.

Needle aspiration of calcium deposits (barbotage) is a frequently used treatment for RCCT (Figure 2). Washing by ultrasound-guided barbotage (UGB) has superior results than corticosteroids, even for calcium deposits of >5 mm [29]. However, some patients experience persistent or recurrence symptoms probably due to associated rotator cuff injuries [29, 30].

**Figure 3:**
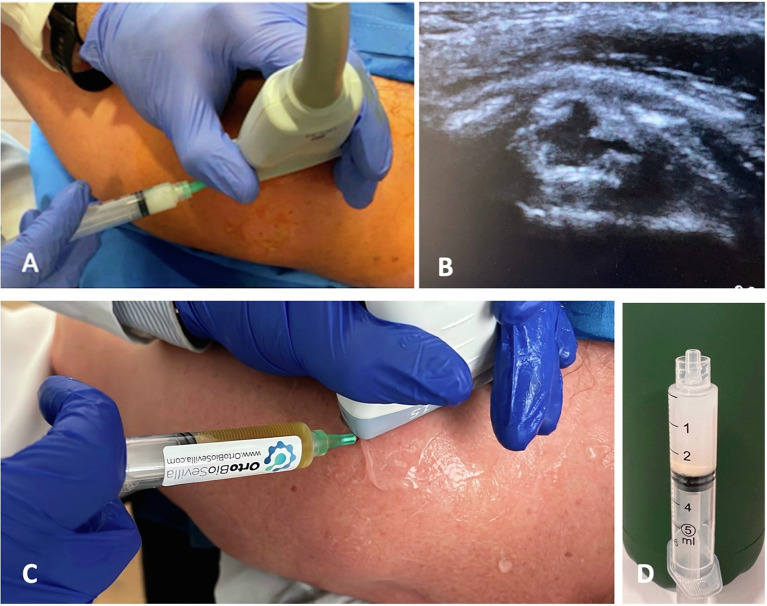
(A) Calcium aspiration in a syringe. (B) Ultrasound image of the sign of Nidus (break in the thickness of the supraspinatus (long axis). (C) Infiltration with intralesional PRP (3mL in nidus) and 6 mL in the bursa. (D) An image showing a syringe with the calcium deposits after the barbotage technique.

For this reason, in associated tears of the rotator cuff, it would be advisable to add to UGB an intralesional injection of plasma rich platelets [31] (Figure 3). Some studies have shown that platelet-rich plasma injections are effective in pain associated with chronic tendinopathy [32–34].

Pakos et al. [35] proposed the combination of calcific deposit needling with “shock waves”, but in reality there is a conceptual error in their study since what they actually applied were low-energy radial pressure waves. This is not the technology of first indication as explained above and it is likely that they have contributed little to the final result.

## Surgical decision making

While many cases can be managed conservatively with physical therapy, nonsteroidal anti-inflammatory drugs (NSAIDs), corticosteroid injections, ESWT, and mini-invasive procedures, surgery becomes a consideration when these measures fail [1, 18].

Typically, surgery is recommended for patients who have not achieved satisfactory relief from symptoms after at least 6 months of non-surgical management [36]. Additionally, surgery is indicated in cases where the calcific deposits are large or have resulted in significant mechanical impingement, limiting the range of motion and causing mechanical symptoms such as catching or locking [37].

Several surgical techniques can be employed, including arthroscopic debridement and removal of calcific deposits, as well as subacromial decompression to address associated impingement [38].

Prognostic factors influencing the outcome of surgical intervention in rotator cuff calcific tendinitis are multiple. Patient’s age, size and density of the calcific deposits, and duration of symptoms are significant predictors of surgical success. Younger patients tend to have better outcomes due to their generally superior healing capacity [38]. The morphology of the calcific deposits also plays a critical role; dense, well-defined deposits are easier to remove completely, which is associated with better postoperative results [1].

The chronicity of symptoms is another important prognostic factor. Patients with shorter symptom durations before surgery tend to experience better outcomes compared to those with long-standing symptoms, possibly due to less extensive tendon degeneration and inflammatory changes [39]. Moreover, the presence of concomitant shoulder pathologies, such as rotator cuff tears or significant subacromial bursitis, can negatively impact surgical outcomes and should be meticulously evaluated preoperatively [40].

Overall, the decision to proceed with surgery in cases of rotator cuff calcific tendinitis should be individualized, taking into account the severity of symptoms, failure of conservative treatments, and the patient’s overall health and activity level. With appropriate patient selection and surgical technique, the prognosis for recovery from rotator cuff calcific tendinitis is generally favorable, with many patients achieving significant pain relief and improved shoulder function [41].

## Surgical treatment

Regarding the surgical management of RCCT, the first point to be highlighted is that such surgery is an exception. Conservative management always plays a significant role, and shall be thoroughly exhausted before any surgical intervention is considered [1, 18].

When it comes to surgery, although high-level evidence studies have not found significant differences in outcomes between different open and arthroscopic procedures [42], the arthroscopic procedure is the most indicated surgical technique.

Even though the main surgical goal is to remove the calcific deposits in the rotator cuff, a usual and systematic arthroscopic evaluation of the shoulder must be performed. This arthroscopic analysis includes identyfing rotator cuff tears and long head of the biceps tears and treating them, if necessary.

Removal of calcific deposits, combined with appropriate rotator cuff repair, if necessary, results in significant pain relief and functional improvement. Hashiguchi et al. reported that the mean shoulder score significantly improved from 69.7 points, before surgery, to 97.8 points at the final follow-up, with most patients achieving complete pain relief and no recurrence of calcific deposits [43]. Similarly, Wilson and Field observed that meticulous removal of calcific deposits, coupled with appropriate rotator cuff repair techniques, resulted in excellent outcomes, with patients experiencing significant improvements in shoulder function and reduction in pain [44].

The initial key point is to properly identify the exact location of the calcific deposit. Most calcific deposits occur in the supraspinatus and infraspinatus tendons. These deposits are usually identified with the arthroscope in the subacromial space, after a broad subacromial bursectomy is performed. It is wise to slowly internally and externally rotate the shoulder, as this maneuver facilitates the recognition of the calcific deposit. The use of a hypodermic needle, or equivalent, is recommended for accurate localization of the calcific deposits, which can be particularly helpful when the deposits are not immediately visible [45].

**Figure 4:**
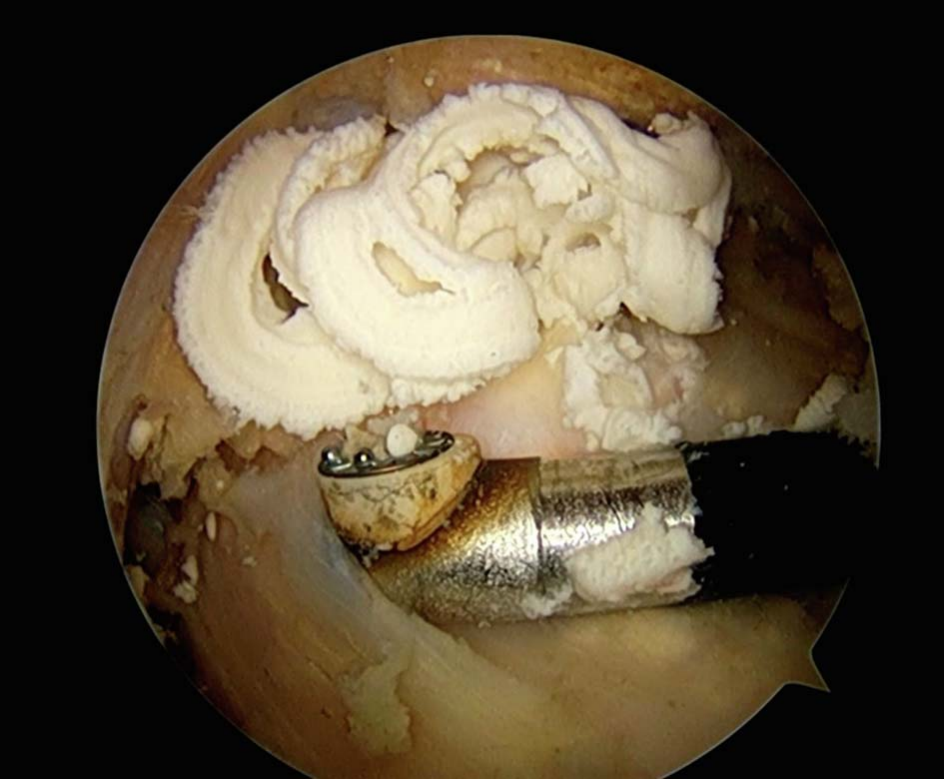
Arthroscopic view performing blunt compression of the calcification after opening the supraspinatus superficial layer.

**Figure 5:**
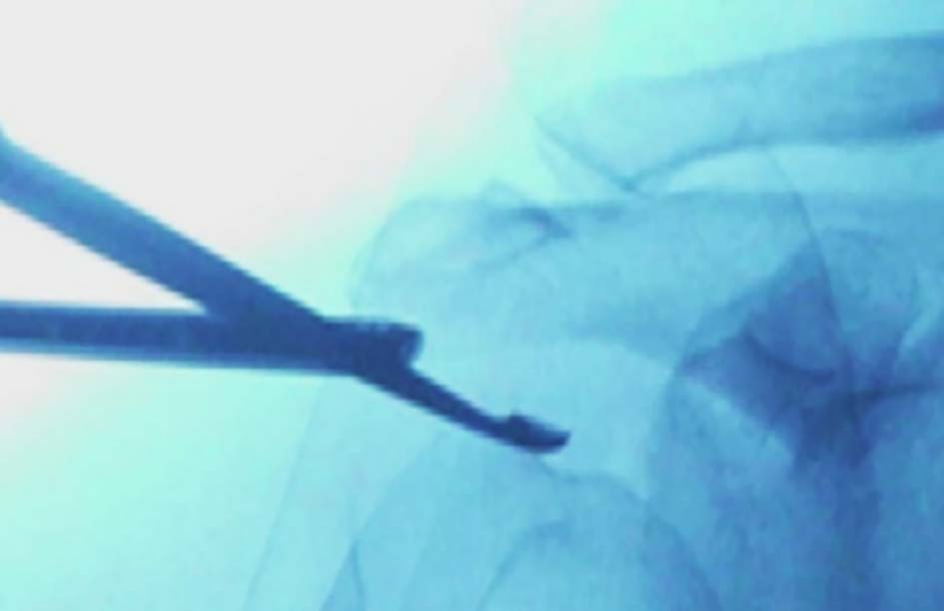
Radioscopic view of a curette and an arthroscope on C-ARM during the treatment of calcific tendonitis in the supraspinatus tendon.

After identifying the calcific deposit, opening of the calcification is typically performed using a small scalpel, creating a longitudinal incision over the deposit. The calcification can then be resected either by compression, where a blunt probe is used to express the calcific material (Figure 4), or by curettage, and the use of a shaver to meticulously remove the deposits [2]. The use of radioscopy (C-ARM) is also advisable to ensure that all calcific deposits have been broadly removed (Figure 5) [46].

There is a debate in the literature regarding the necessity of performing an acromioplasty during this procedure. Some authors advocate for acromioplasty to relieve subacromial impingement, which is often associated with calcific tendonitis. However, others argue that acromioplasty may not be necessary, as the primary issue is the calcification itself rather than structural impingement. Studies have shown mixed results, with some indicating no significant difference in outcomes with or without acromioplasty [47].

Another area of controversy is whether to perform a total or partial resection of the calcific deposit [43–45]. In some cases, a partial resection might be considered, especially if the deposit is deeply embedded in the tendon and its complete removal could cause extensive damage to the rotator cuff. Hashiguchi et al. reported satisfactory outcomes even when some residual calcific deposits remained, if most of the deposit was excised [43]. The spontaneous reabsorption of residual calcifications in the postoperative period has been documented in various studies. Maier et al. observed that residual calcifications reabsorb spontaneously within the first few months after surgery. This process is typically accompanied by significant clinical improvement, with patients reporting reduced pain and increased shoulder function [45].

Additionally, there is debate on whether to repair, or not, the resulting rotator cuff defect after removing the calcific deposit. Depending on the size of the deposit, a rotator cuff tear may sometimes be created because of the removal. This is not uncommon, and when it occurs, such rotator cuff tears traditionally were repaired, usually with anchors [43–45]. However, another approach is to minimize the resection and leave partial-thickness rotator cuff defects unrepaired. Wilson and Field suggested that such defects often do not require repair and can be left to heal naturally [44]. This approach minimizes surgical morbidity and preserves as much of the rotator cuff tissue as possible, which can be beneficial for patients, minimizing the risk for complications.

## Conclusion

Rotator cuff calcifications are a highly prevalent finding in asymptomatic population. Its etiopathogenesis is not completely clear, but there is the possibility that a subgroup of patients is influenced by hormonal disorders.

When the presence of the calcific deposits on images is associated with symptoms, the implementation of treatment is justified. Each of the therapeutic options has advantages and disadvantages (Tables 2 and 3). The ideal is to start with rehabilitation unless there are poor prognostic factors for it. In the rest of the cases, or if there is no clinical response, the ideal option are focused shock waves because it is a non-invasive modality, with a low complication rate. The next stage of treatment are mini-invasive procedures or surgery.

**Table 2:** Main indications for each method.

Treatment	Indication
Conservative treatment	First-line approach.
ESWT	After rehabilitation failure. Low-risk alternative with outcomes comparable to surgical procedures.
Barbotage	Large or persistent calcific deposits. Most effective in patients with calcific tendinopathy during the formative and resorptive phases.
Surgery	Indicated for patients with severe pain and significant functional impairment that persists despite thorough management for at least six months.

**Table 3:** Comparison of results with different techniques.

Author	Treatment	Type of study	*N*	Success rate
Ogon et al. [20]	Conservative	Prospective cohort study	420	73%
Moya et al. [23]	ESWT	Retrospective series	23	82.6%
Werry et al. [30]	Barbotage	Retrospective chart review	179	Not statistically significant at 1 year
Vassalou et al. [36]	Barbotage	Prospective series	79	Results influenced by deposit size and initial pain level at 1 year
Cho et al. [41]	Artrhoscopy	Retrospective series	35	Clinical scores improved slowly, recovery of shoulder function and pain relief required up to 6 months
Balke et al. [47]	Artrhoscopy	Case series	70	Operated shoulders have significantly lower clinical scores than healthy shoulders.

## Data Availability

Data sharing is not applicable to this article as no data sets were generated or analyzed during this study.
